# Supplementary material for: Simultaneous profiling of RNA isoforms and chromatin accessibility of single cells of human retinal organoids
Source: Nat Commun. 2024 Sep 13;15:8022. doi: 10.1038/s41467-024-52335-0 (PMC11399327; doi:10.1038/s41467-024-52335-0)
Supplement: Supplementary file 1 — Supplementary Information [file 41467_2024_52335_MOESM1_ESM.pdf]

Supplementary Information

Supplementary Figures

Supplementary Fig. 1. Overview of scRICA-seq in low throughput.
Supplementary Fig. 2. Molecular structure of DNA oligos and the chemistry for the workflow in high throughput.
Supplementary Fig. 3. Molecular structure of DNA oligos and the chemistry for the workflow in low throughput.
Supplementary Fig. 4. scRCAT-seq2 cDNA and library array size distribution.
Supplementary Fig. 5. Pearson correlation comparing consistency of gene expression levels in replicated samples of hESC (a) and HEK293T (b).
Supplementary Fig. 6. Performance of scRCAT-seq2.
Supplementary Fig. 7. Detection of frozen tissue using scRCAT-seq2 (mouse kidney).
Supplementary Fig. 8. The single-cell nucleus acquisition process of the 45-day cultured human retinal organoids and the quality inspection analysis of scRNA seq and scATAC seq.
Supplementary Fig. 9. Whole gene read coverage map of HES1 gene captured by scRCAT-seq2 sequencing in the D45 sample.
Supplementary Fig. 10. Heatmap displaying marker genes of each cluster detected by scRCAT-seq2.
Supplementary Fig. 11. Relationship between differential gene expression and chromatin accessibility during RPC differentiation into cones and RGCs.
Supplementary Fig. 12. Relationship between differential isoform expression and promoter accessibility of key genes during RPC differentiation into cones.
Supplementary Fig. 13. Analysis of the relationship between chromatin accessibility and gene expression of transcription factor-regulated target genes.
Supplementary Fig. 14. The correlation between significant changes in chromatin accessibility and gene expression in comparison between cone and RPC, involving different transcription factors as target genes.
Supplementary Fig. 15. Correlation of RNA isoform choice with chromatin accessibility and TFs during RPC development.
Supplementary Fig. 16. Analysis of motif enrichment at variable splicing sites with different levels of chromatin accessibility.
Supplementary Fig. 17. Analysis of the relationship between chromatin accessibility and gene expression of key transcription factors and splicing factors regulating isoform splicing events.

Supplementary Figures

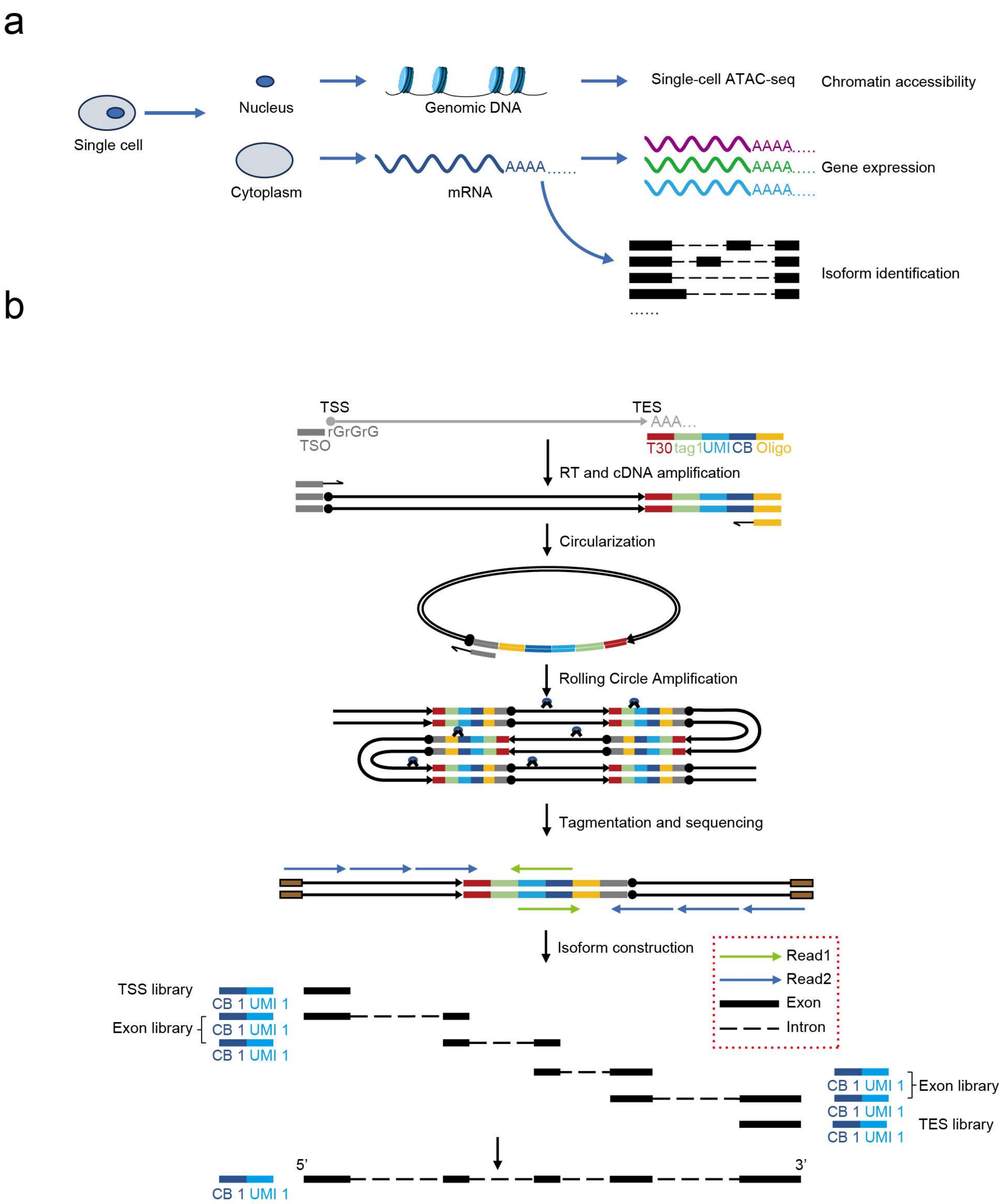

Supplementary Fig. 1. Overview of scRICA-seq in low throughput. a Schematic illustration of scRICA-seq to simultaneously profile the RNA isoforms and chromatin accessibility for the same single cell, by integrating the scRNA-seq, scATAC-seq, scRCAT-seq2. **b** Schematic illustration of scRCAT-seq2 to depict the step-by-step procedure for generating the full-length cDNA library in high throughput. CB: Cell barcode; UMI: Unique Molecular Identifier; TSO: Template-switching oligo; T30: 30 repeating T bases.

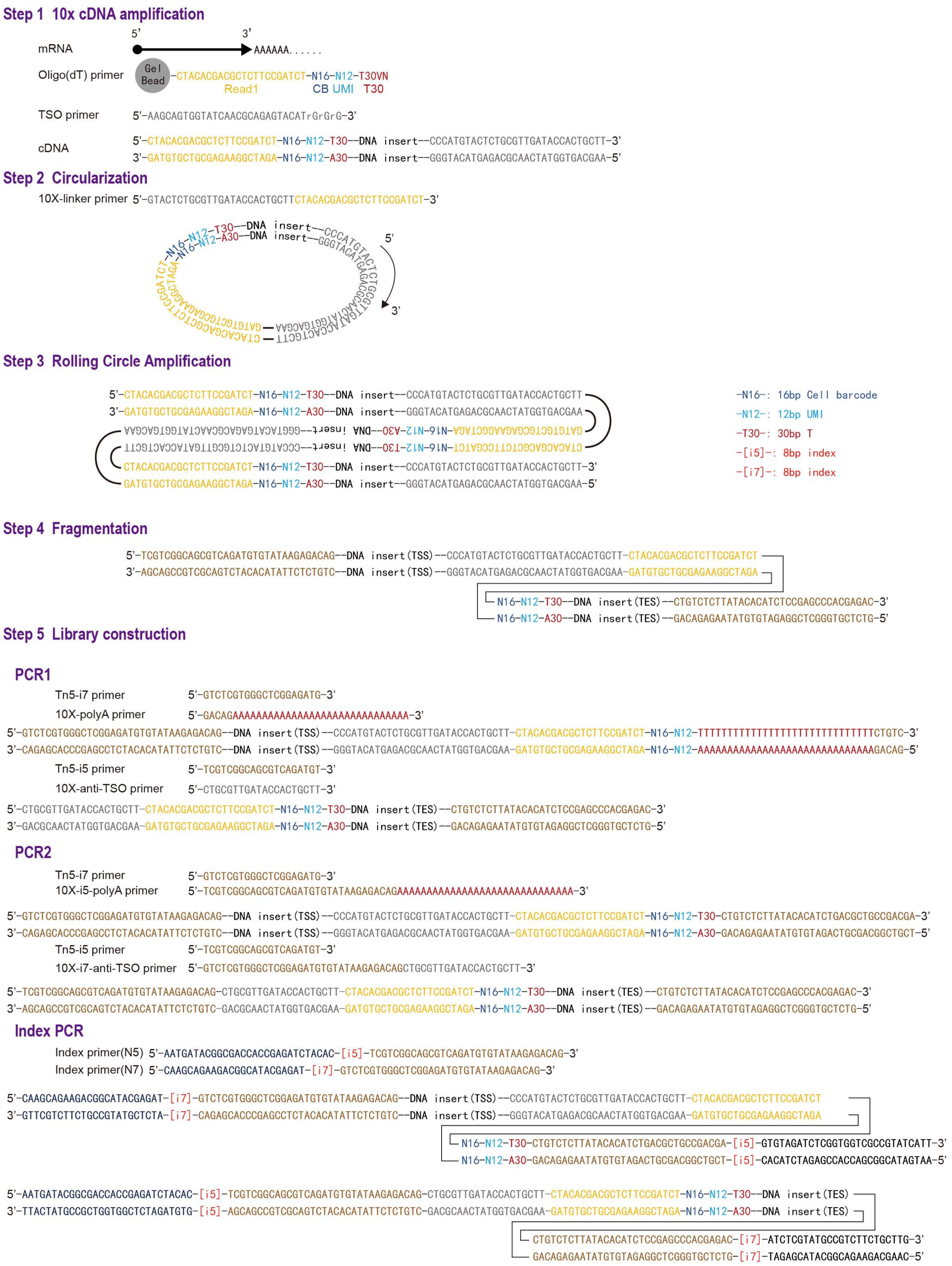

Supplementary Fig. 2. Molecular structure of DNA oligos and the chemistry for the workflow in high throughput.

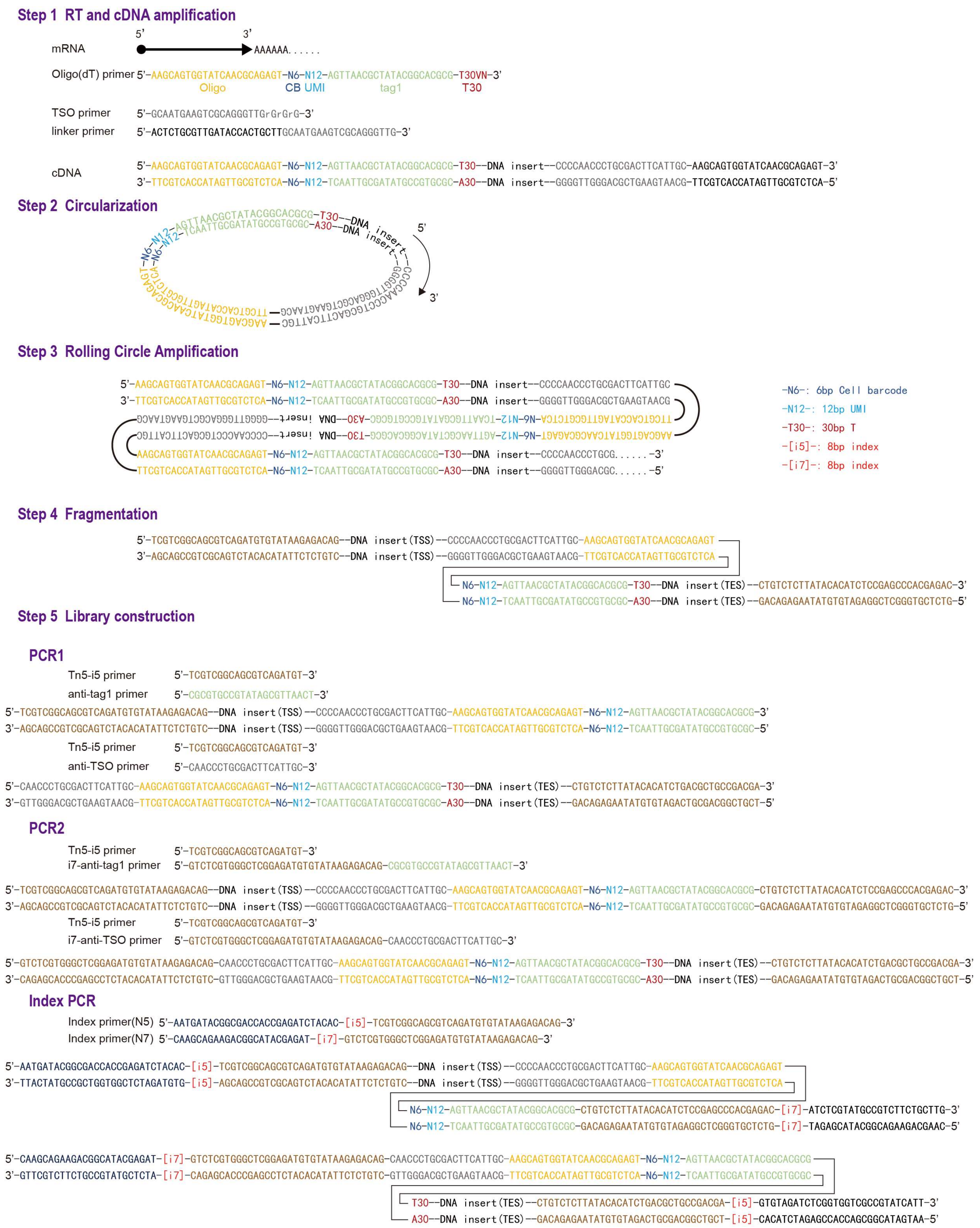

Supplementary Fig. 3. Molecular structure of DNA oligos and the chemistry for the workflow in low throughput.

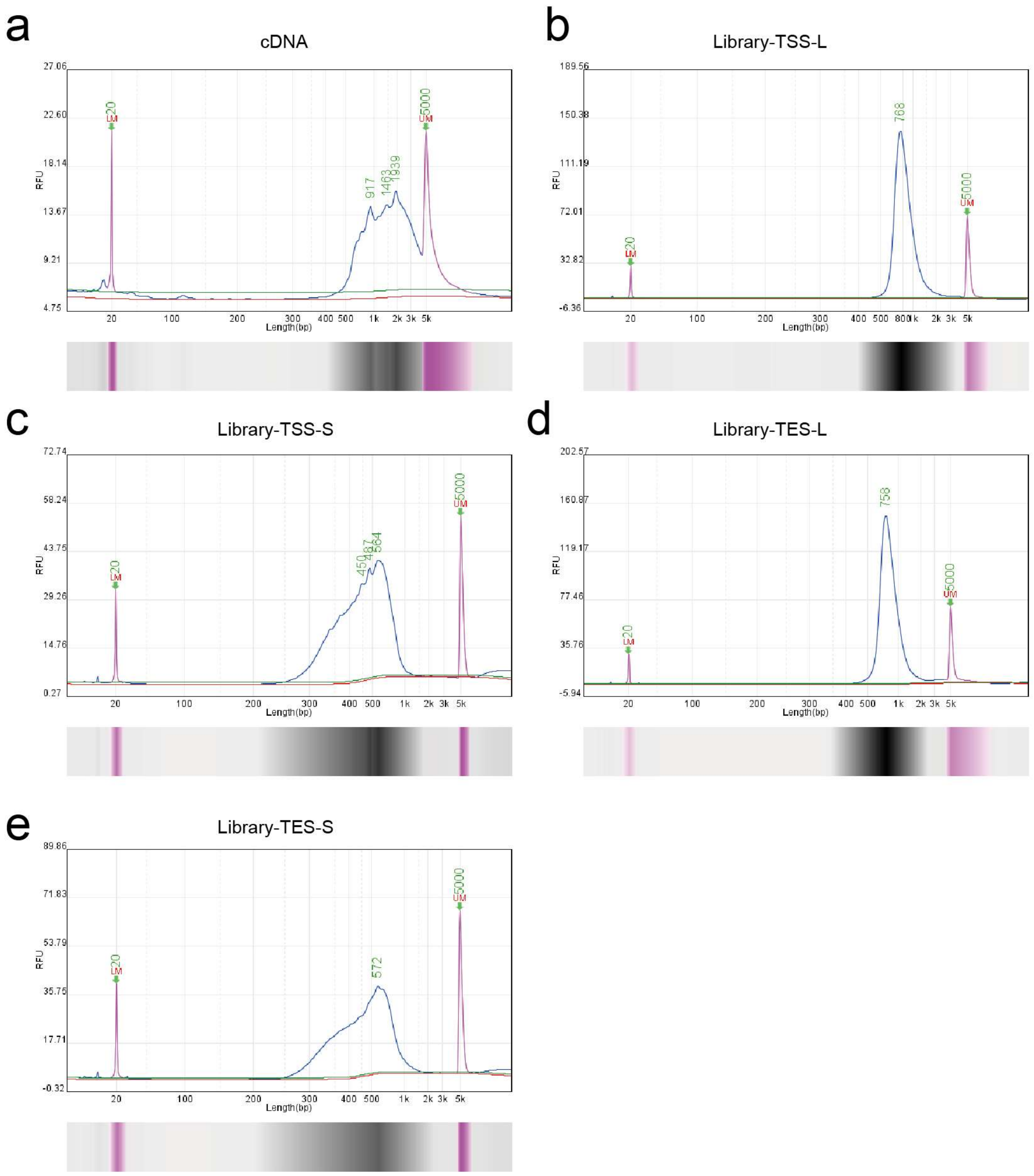

Supplementary Fig. 4. scRCAT-seq2 cDNA and library array size distribution. a cDNA fragment size distribution on the Bioanalyser platform (Qsep100^{TM}). **b-e** Visualization of scRCAT-seq2 library fragment size distribution on the Bioanalyser platform. Library-TSS-L: Long fragments of TSS library; Library-TSS-S: Short fragments of TSS library; Library-TES-L: Long fragments of TSS library; Library-TES-S: Short fragments of TSS library.

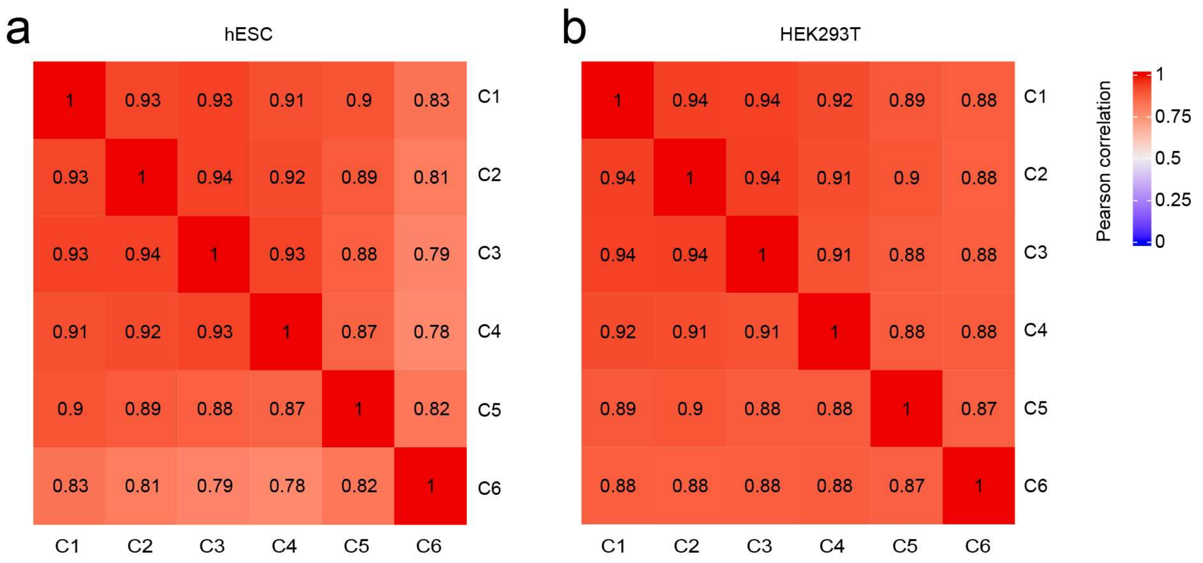

Supplementary Fig. 5. Pearson correlation comparing consistency of gene expression levels in replicated samples of hESC (a) and HEK293T (b). n = 6, A total of 6 replicates of scRCAT seq2 were performed.

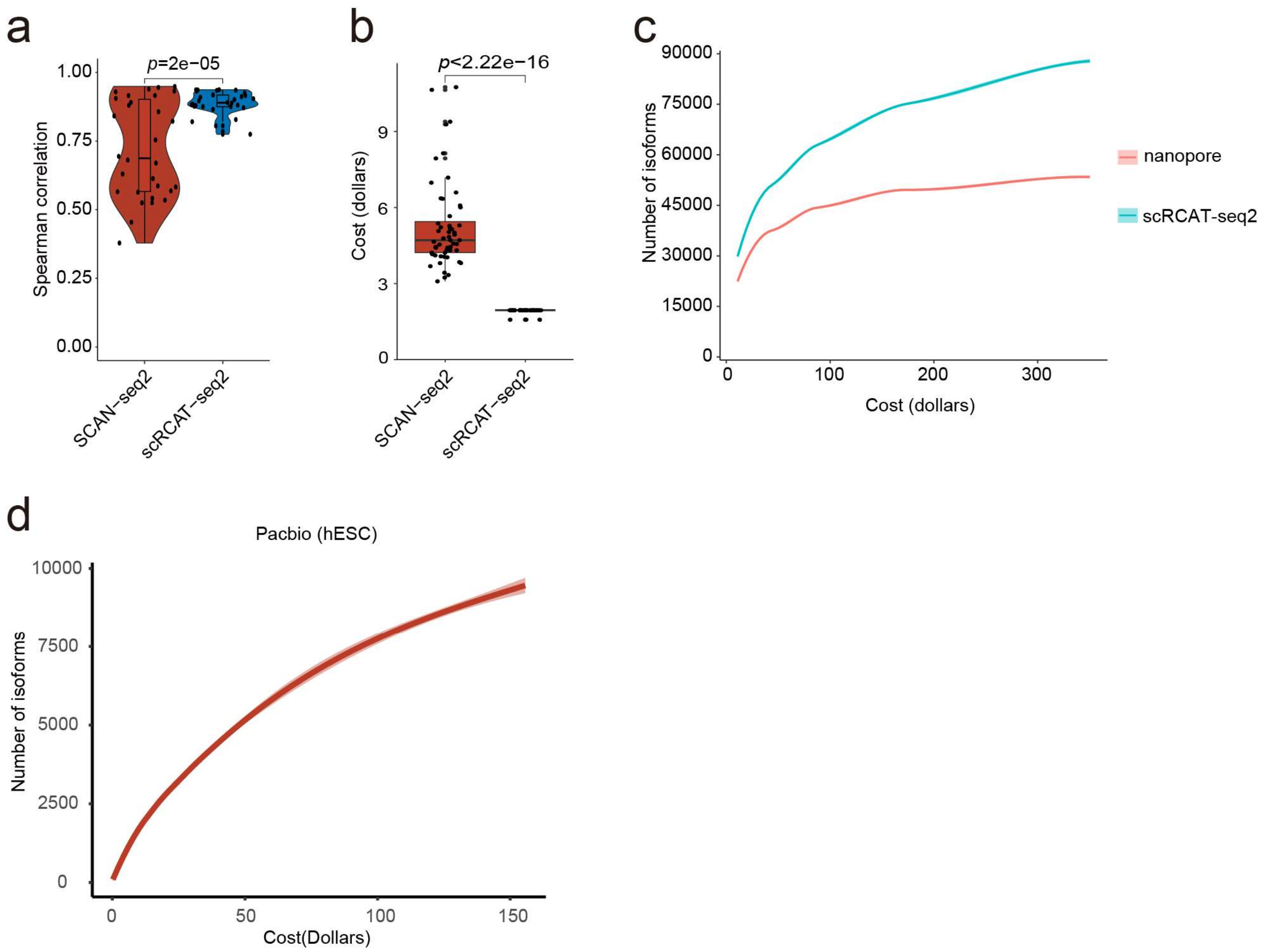

Supplementary Fig. 6. Performance of scRCAT-seq2. a Spearman correlation comparing SCAN-seq and scRCAT-seq2 in different cell correlations (n=36). **b** Comparison of the cost per cell for obtaining saturated data using SCAN-seq (n = 240, A total of 240 K562 cells and 293T cells) and scRCAT-seq2 (n = 12, A total of 12 ES cells and 293T cells). **c** Comparison of isoform counts obtained by scRCAT-seq2 and nanopore at different costs under high throughput. **d** The saturation curve of scISOr seq sequencing. Source data are provided as a Source Data file.

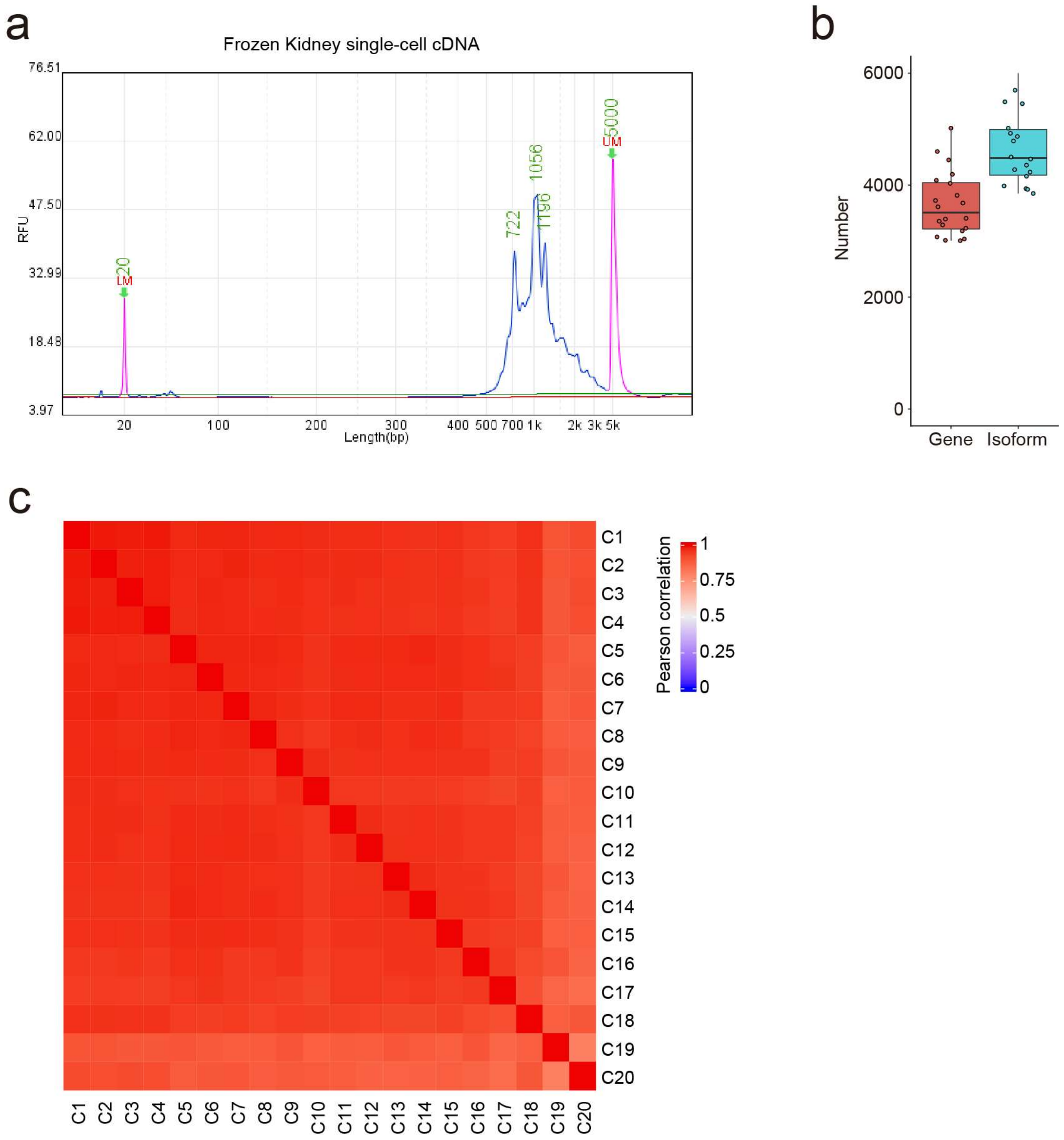

Supplementary Fig. 7. Detection of frozen tissue using scRCAT-seq2 (mouse kidney). a cDNA fragment size distribution on the Bioanalyser platform (Qsep100^{TM}). **b** The number of genes and isoforms captured per cell in mouse kidney cells by scRCAT-seq2 (n = 20, 20 kidney cells). **c** Pearson correlation comparing consistency of gene expression levels in each mouse kidney cell.

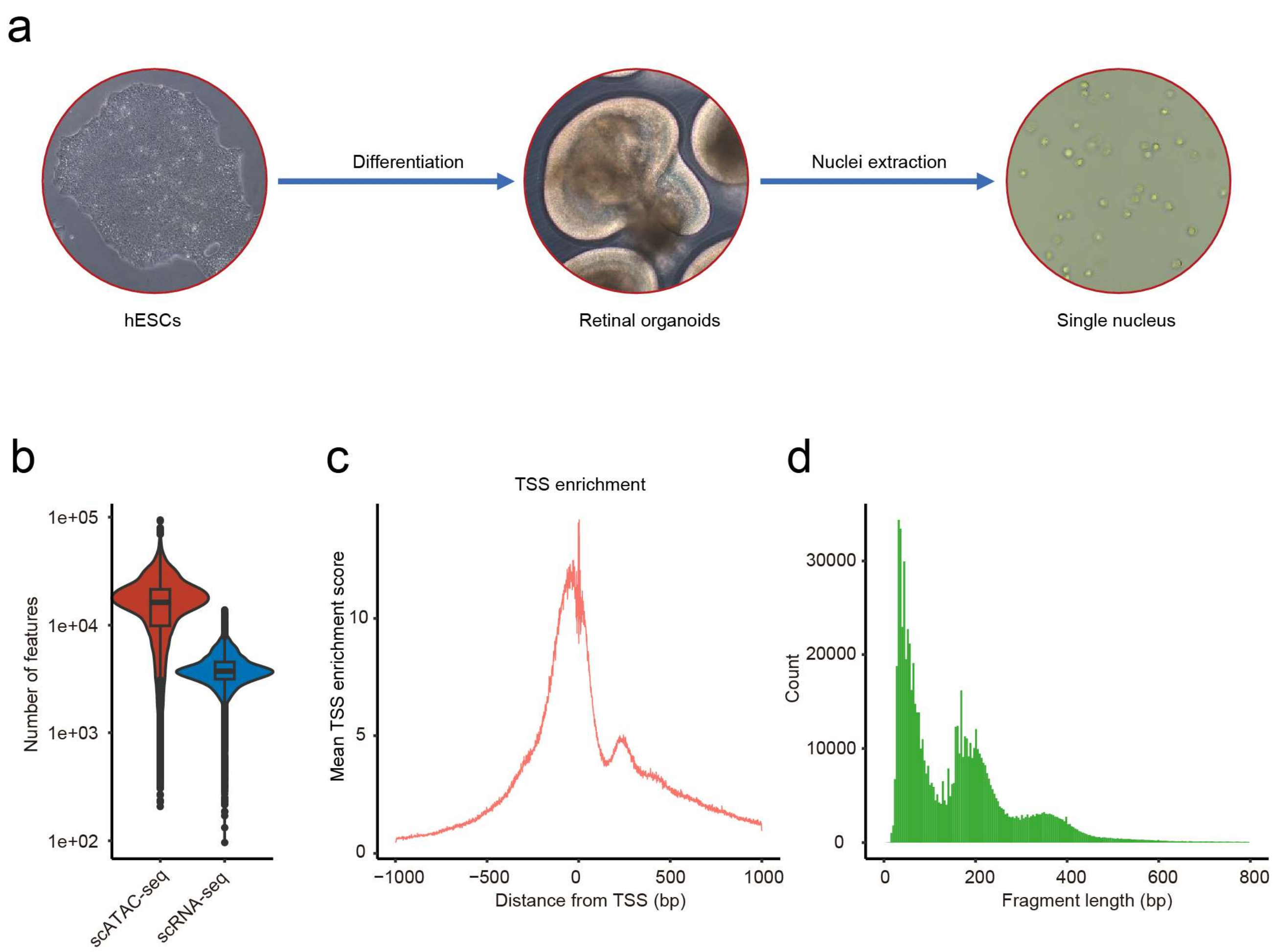

Supplementary Fig. 8. The single-cell nucleus acquisition process of the 45-day cultured human retinal organoids and the quality inspection analysis of scRNA seq and scATAC seq. a Schematic illustration of organoid preparation and sample processing. Starting with culturing and differentiating hESCs into retinal organoids, followed by digestion to obtain single-cell suspensions, and the extraction of cell nuclei for library construction. **b** Vinlon plot illustrating the distribution of the number of genes and peaks per cell. **c** TSS enrichment plot demonstrating the enrichment of scATAC-seq fragments at transcription start sites (TSS). **d** Distribution of scATAC-seq fragment lengths in D45 retinal organoid samples. The histogram displays the periodic signal of nucleosomes.

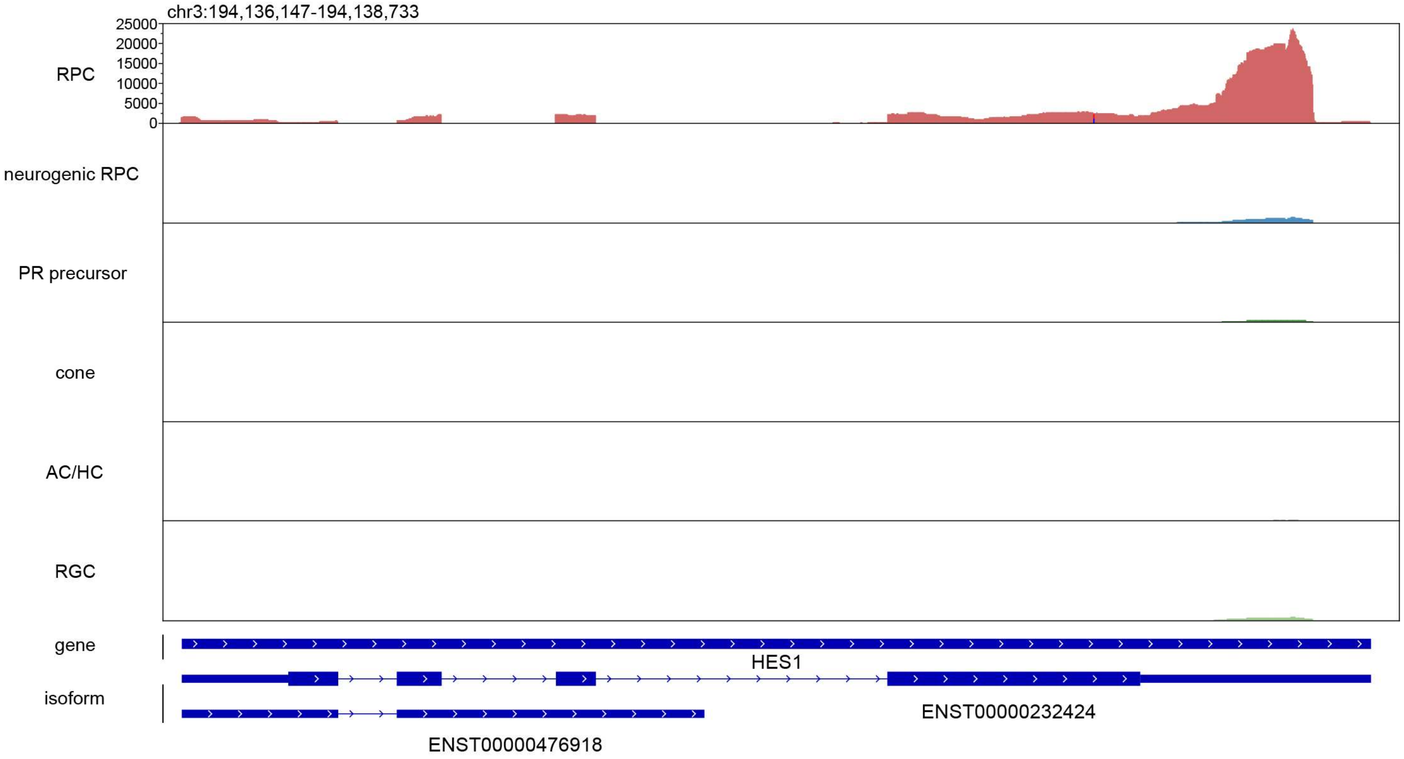

Supplementary Fig. 9. Whole gene read coverage map of *HES1* gene captured by scRCAT-seq2 sequencing in the D45 sample.

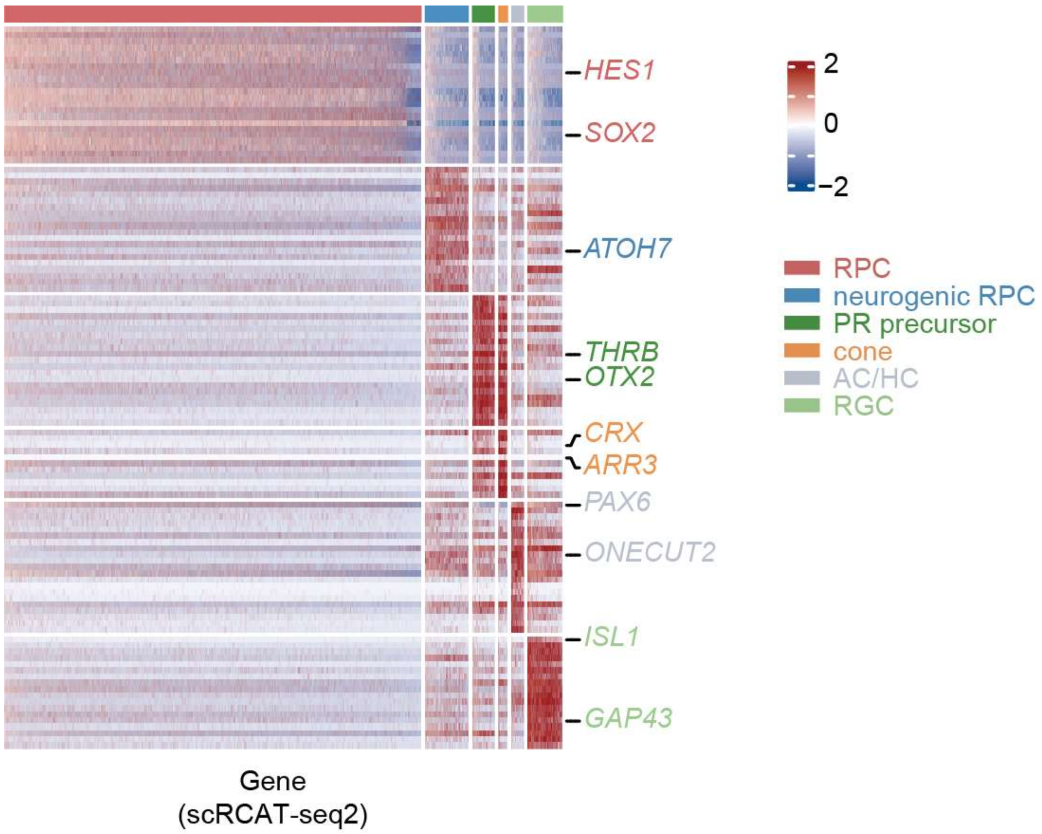

Supplementary Fig. 10. Heatmap displaying marker genes of each cluster detected by scRCAT-seq2.

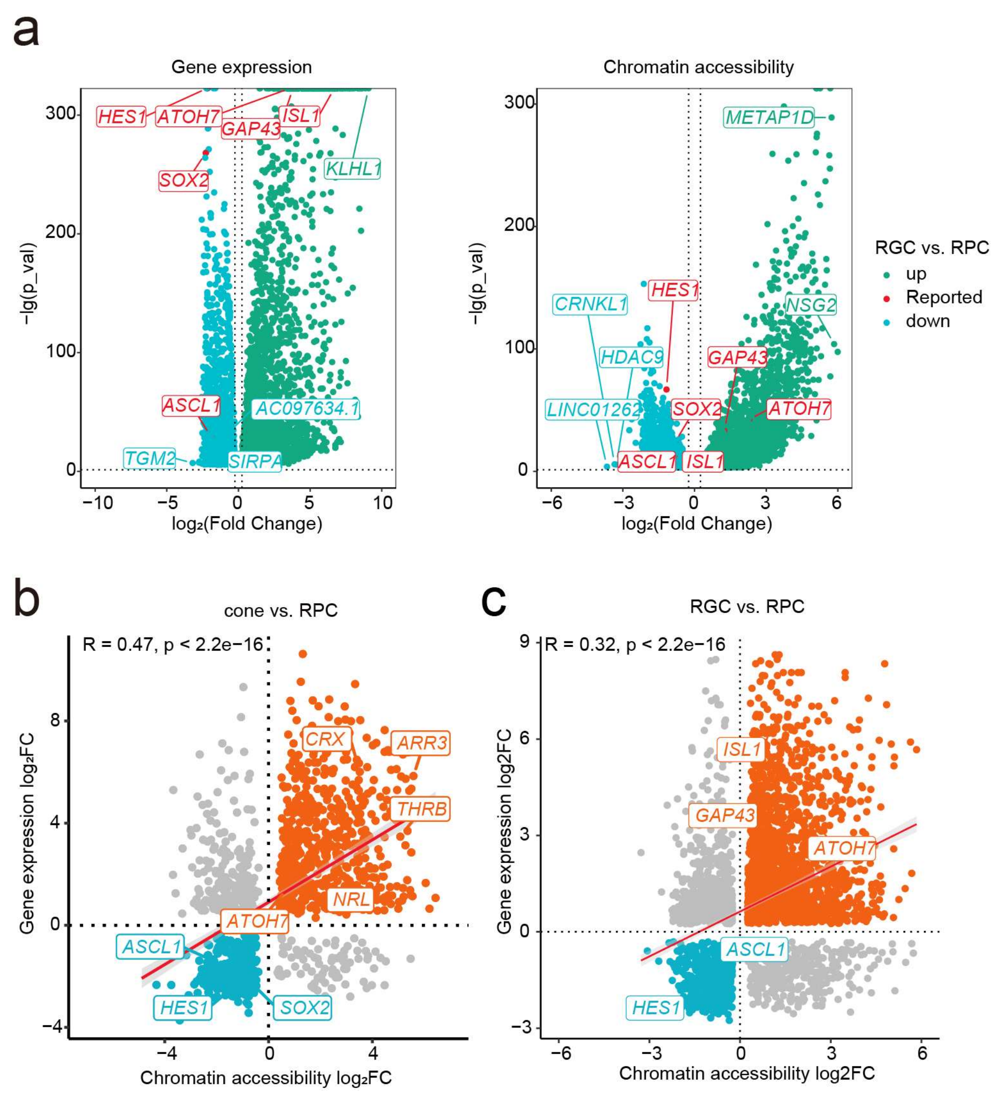

Supplementary Fig. 11. Relationship between differential gene expression and chromatin accessibility during RPC differentiation into cones and RGCs. a Volcano plot showing genes with concordant changes in expression (left) and chromatin accessibility (right) during RPC-RGC development. P-values were calculated using a two-sided Wilcoxon rank sum test. **b** Correlation of gene expression with chromatin accessibility during RPC-Cone development. **c** Correlation of gene expression with chromatin accessibility during RPC-RGC development. Source data are provided as a Source Data file.

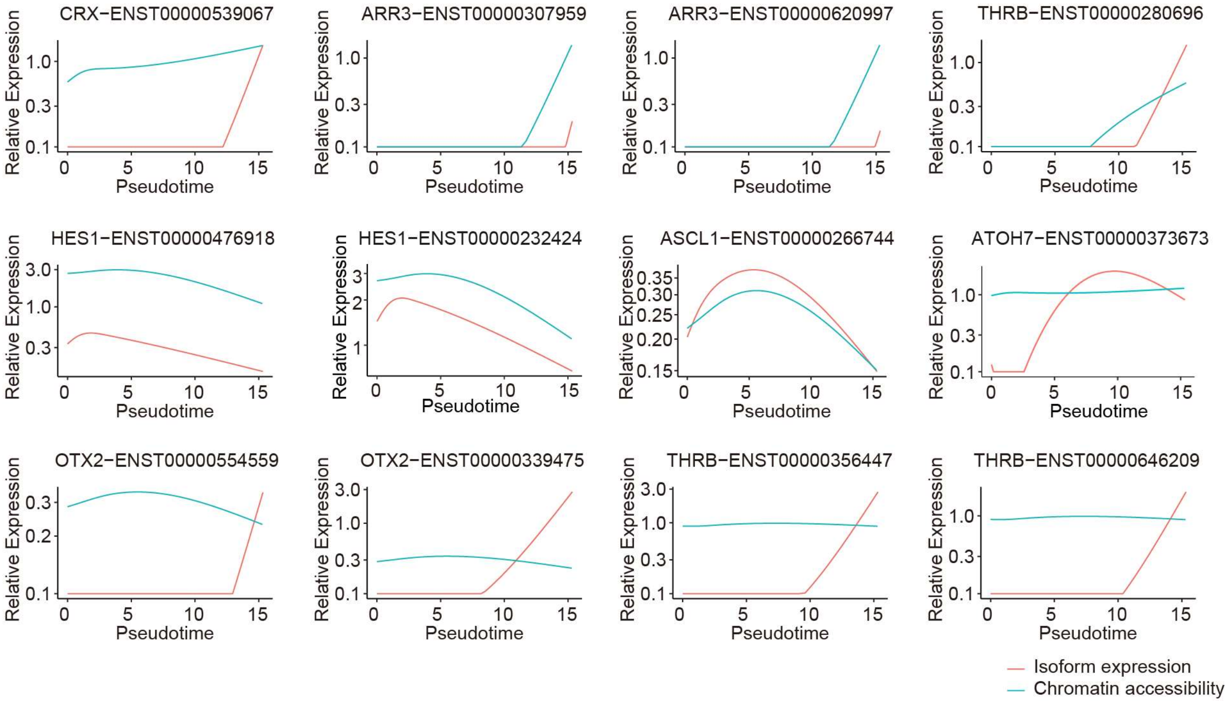

Supplementary Fig. 12. Relationship between differential isoform expression and promoter accessibility of key genes during RPC differentiation into cones.

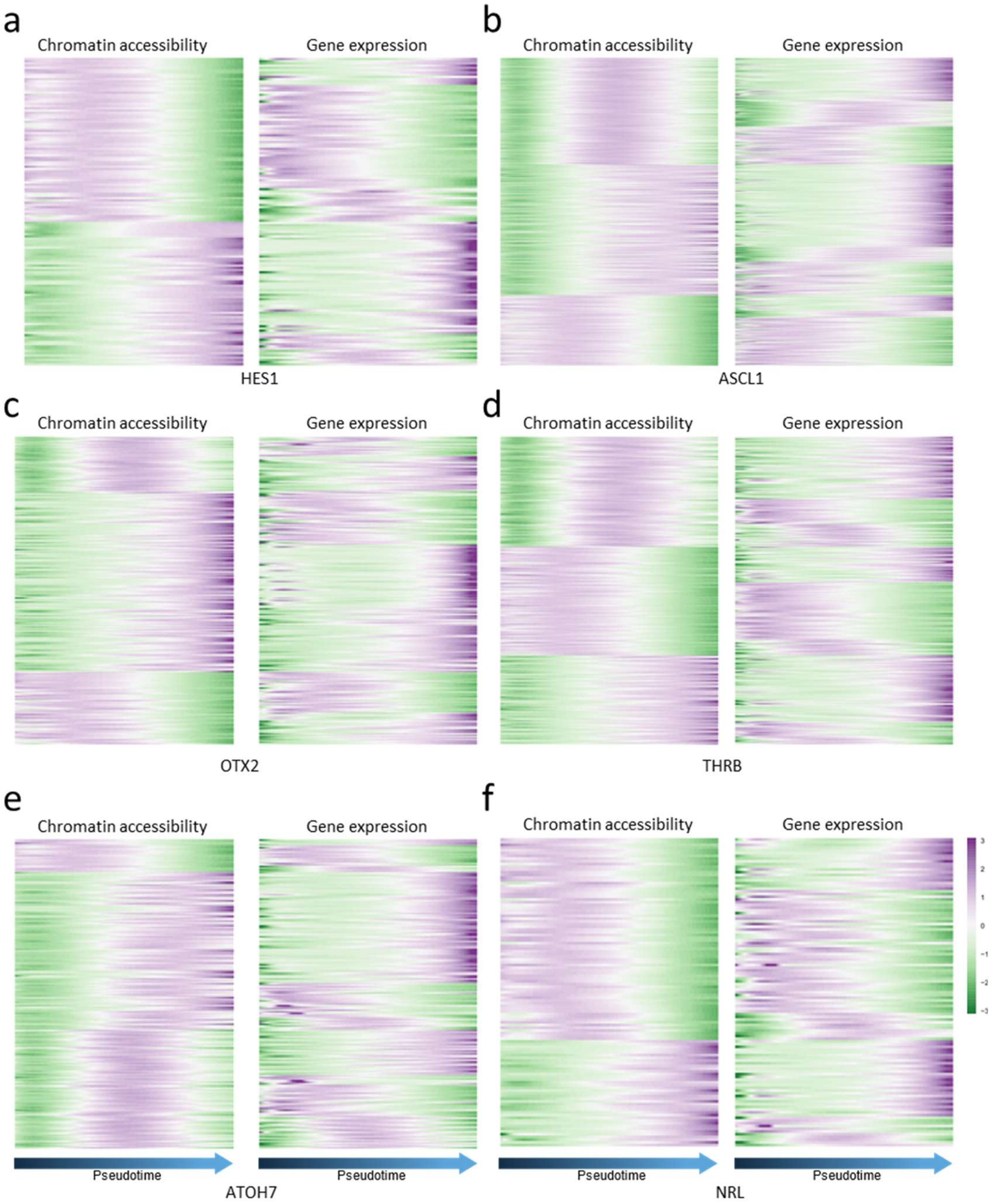

Supplementary Fig. 13. Analysis of the relationship between chromatin accessibility and gene expression of transcription factor-regulated target genes. a-f Changes in chromatin accessibility and gene expression on the RPC differentiation trajectory of downstream target genes regulated by transcription factors HES1 (**a**), ASCL1 (**b**), OTX2 (**c**), THRB (**d**), ATOH7 (**e**), and NRL (**f**). Source data are provided as a Source Data file.

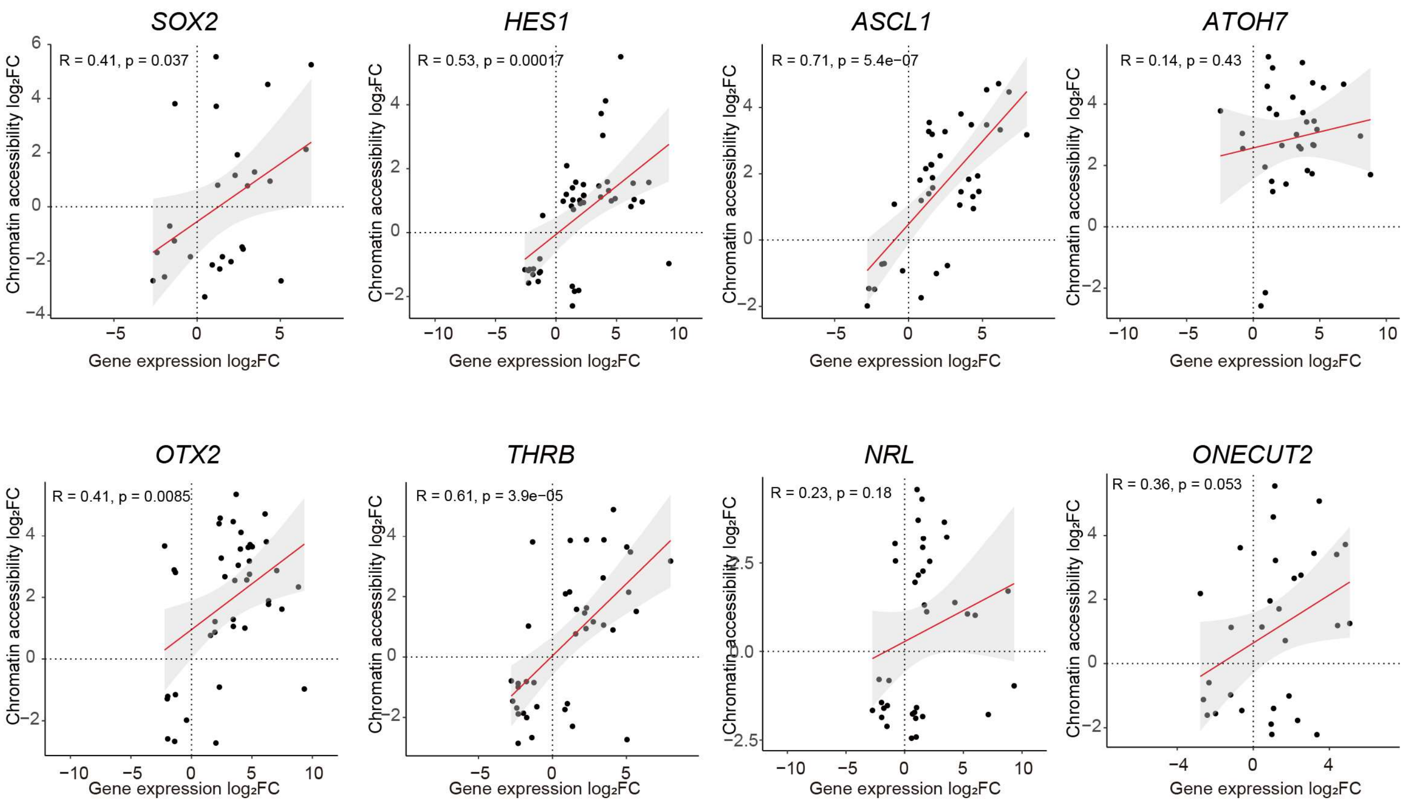

Supplementary Fig. 14. The correlation between significant changes in chromatin accessibility and gene expression in comparison between cone and RPC, involving different transcription factors as target genes. Source data are provided as a Source Data file.

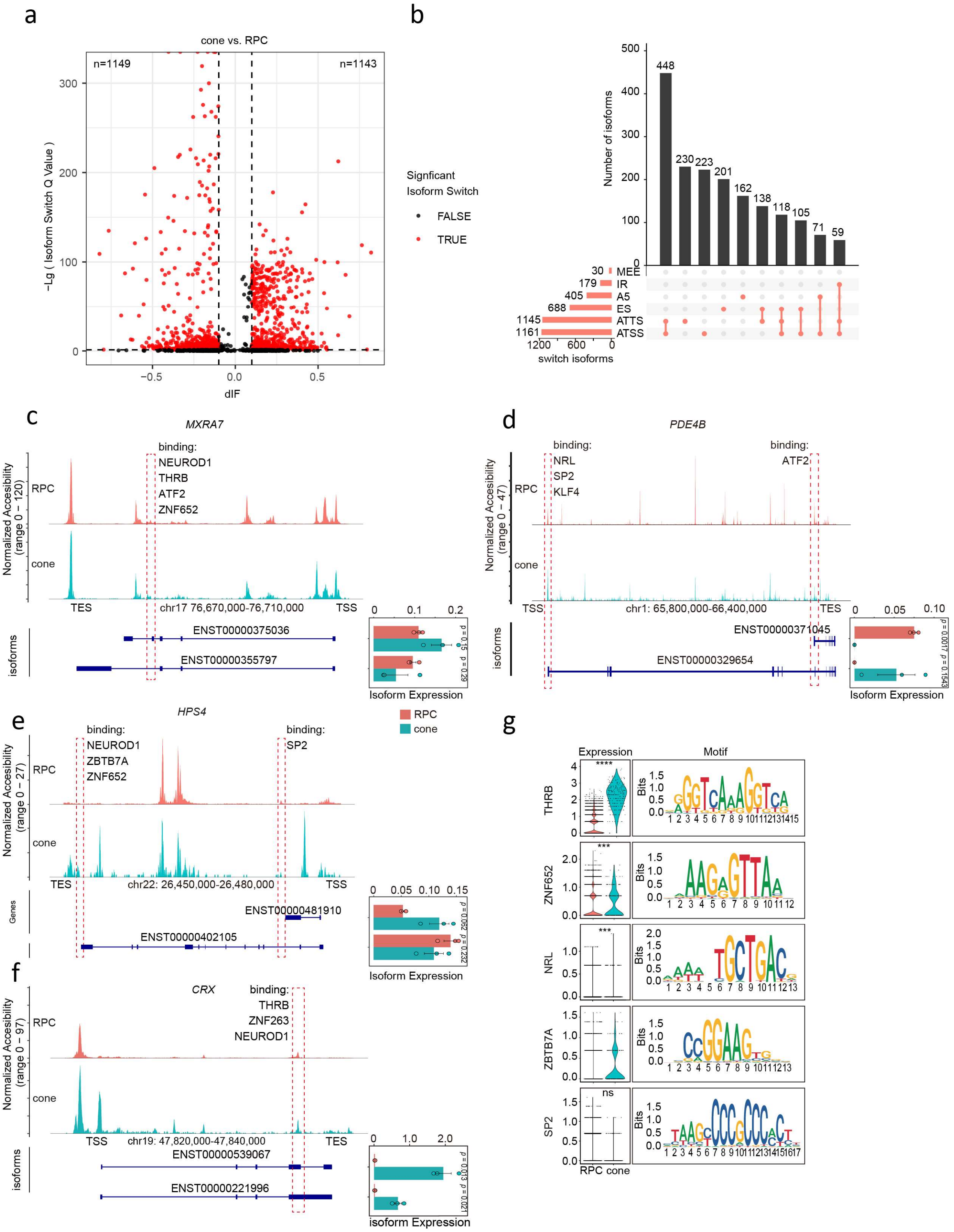

Supplementary Fig. 15. Correlation of RNA isoform choice with chromatin accessibility and TFs during RPC development. a Volcano plot comparing differential isoform changes between cones and RPCs. **b** Intersection analysis of different types of splicing events occurring on the same pair of isoforms. **c** Schematic representation of the *MXRA7* locus undergoing splicing events with concurrent changes in chromatin accessibility. Significance was computed using two-sided *t*-test. Data are presented as mean values +/- SEM. **d-f** Schematic representation of the *PDE4B* (**d**), *HPS4* (**e**) and *CRX* (**f**) loci undergoing splicing events without concurrent changes in chromatin accessibility. Significance was computed using two-sided *t*-test. Data are presented as mean values +/- SEM (n=3 duplicate samples). **g** Integration of transcription factor expression changes and motif analysis at variable splicing sites in **c-f**. Source data are provided as a Source Data file.

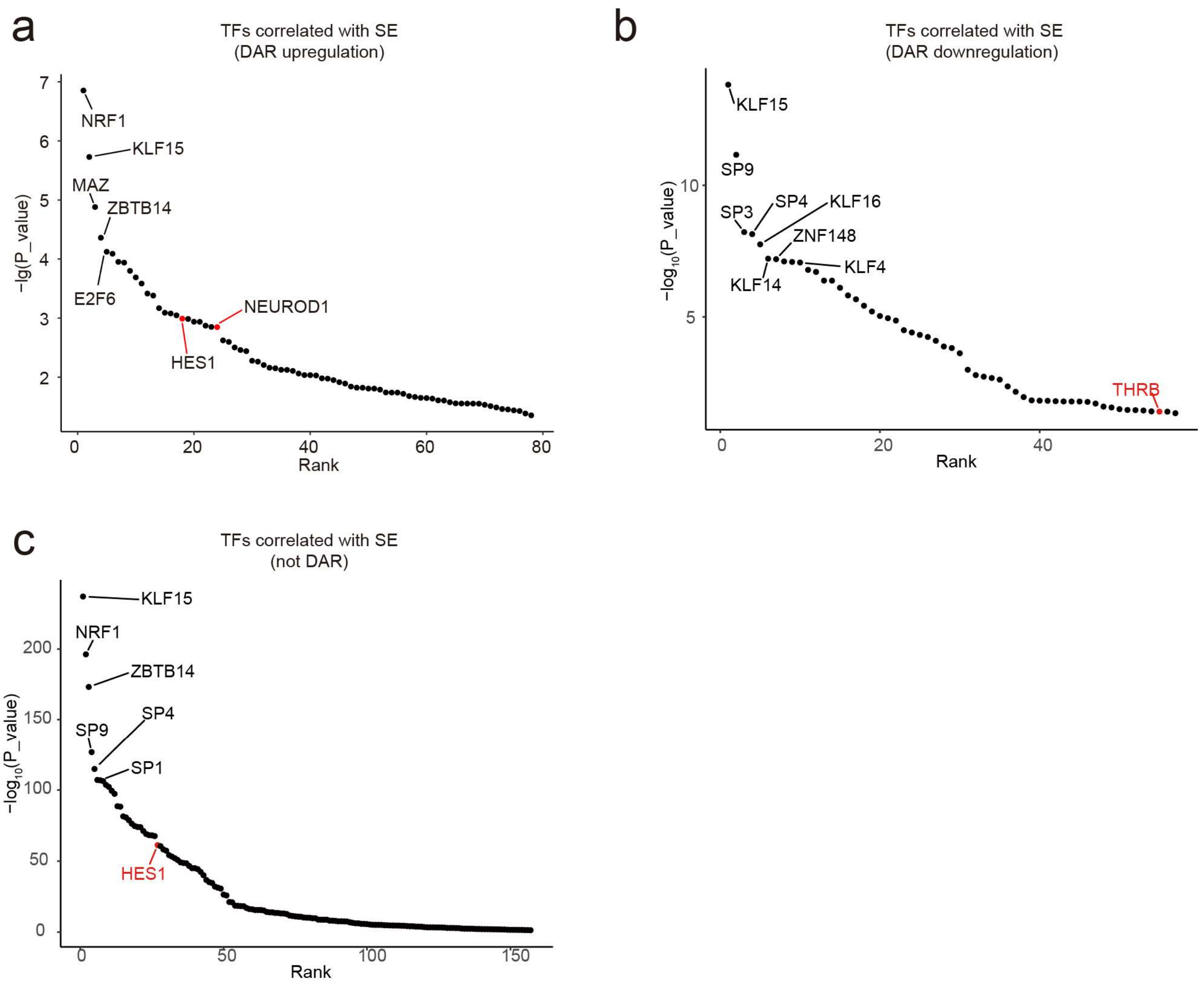

Supplementary Fig. 16. Analysis of motif enrichment at variable splicing sites with different levels of chromatin accessibility. a Enriched TFs motifs with upregulated chromatin accessibility at isoform variable splicing sites (n = 78). **b** Enriched TFs motifs with downregulated chromatin accessibility at isoform variable splicing sites (n = 57). **c** Enriched TFs motifs with no significant changes in chromatin accessibility at isoform variable splicing sites (n = 157). Source data are provided as a Source Data file.

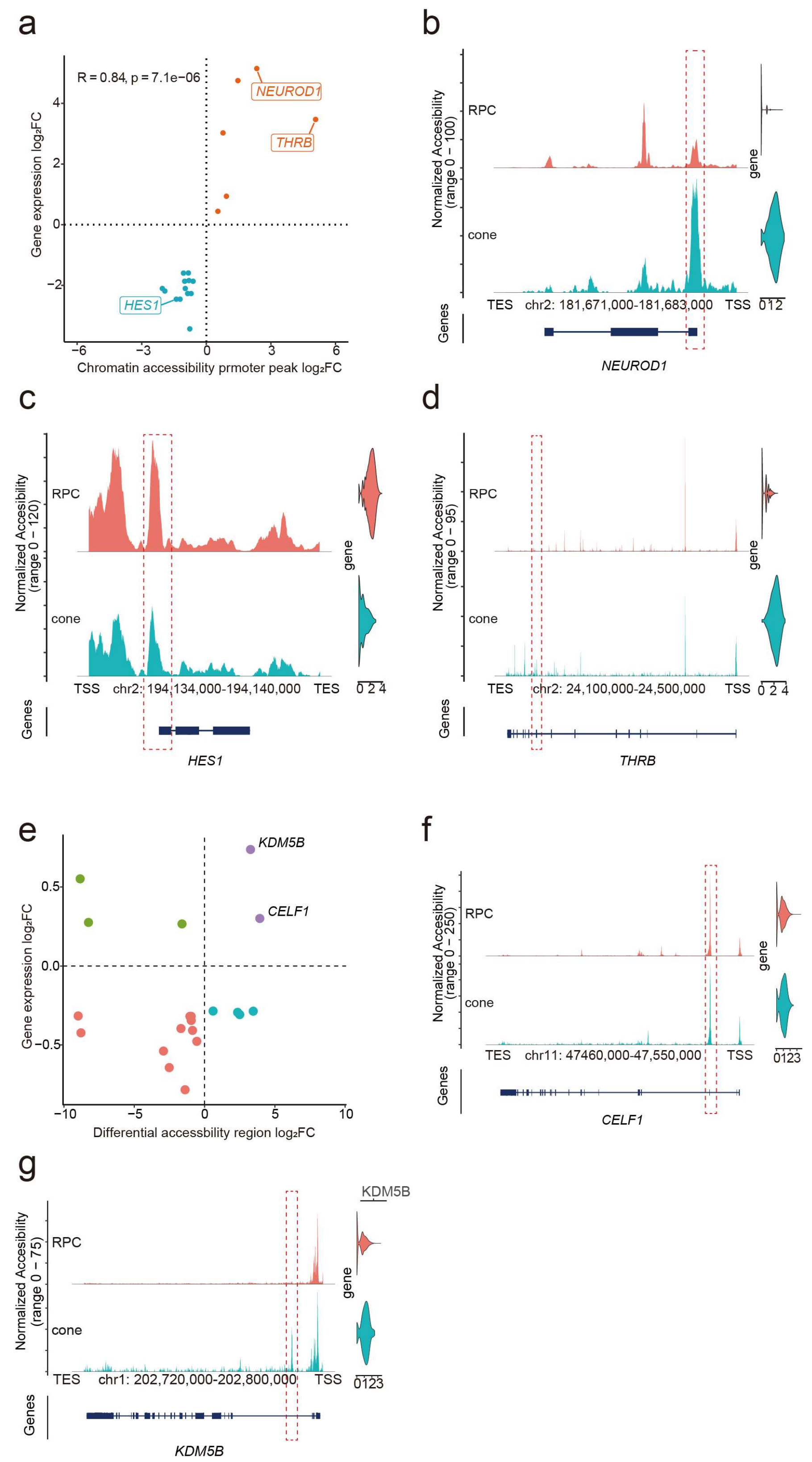

Supplementary Fig. 17. Analysis of the relationship between chromatin accessibility and gene expression of key transcription factors and splicing factors regulating isoform splicing events. a Point plot showing the correlation between chromatin accessibility and gene expression of key transcription factors regulating isoform splicing events. **b-d** Schematic illustration of concordant gene expression and chromatin accessibility alterations in *NEUROD1* (**b**), *HES1* (**c**), and *THRB* (**d**) during RPC-cone development. **e** Point plot showing the correlation between chromatin accessibility and gene expression of key splicing factors regulating isoform splicing events. **f-g** Schematic illustration of concordant gene expression and chromatin accessibility alterations in *CELF1* (**f**) and *KDM5B* (**g**) during RPC-cone development. Source data are provided as a Source Data file.
