# Supplementary material for: Simultaneous profiling of RNA isoforms and chromatin accessibility of single cells of human retinal organoids
Source: Nat Commun. 2024 Sep 13;15:8022. doi: 10.1038/s41467-024-52335-0 (PMC11399327; doi:10.1038/s41467-024-52335-0)
Supplement: Supplementary file 3 — Description of Additional Supplementary Files [file 41467_2024_52335_MOESM3_ESM.pdf]

Description of Additional Supplementary Files

File Name: Supplementary Data 1
Description: Comparison of scRICA-seq and other methods

File Name: Supplementary Data 2
Description: Primer sequence information

File Name: Supplementary Data 3
Description: Reagent information used in the scRCAT seq2
